# Variations in gene expression levels with severity of synovitis in dogs with naturally occurring stifle osteoarthritis

**DOI:** 10.1371/journal.pone.0246188

**Published:** 2021-01-28

**Authors:** Atsushi Yamazaki, Kazuya Edamura, Yuma Tomo, Mamiko Seki, Kazushi Asano

**Affiliations:** Department of Veterinary Medicine, Laboratory of Veterinary Surgery, College of Bioresource and Sciences, Nihon University, Fujisawa, Kanagawa, Japan; University of Messina, ITALY

## Abstract

Osteoarthritis (OA) is one of the major causes of chronic pain in dogs. However, the pathogenesis of OA has not been fully understood in dogs. The objective of this study was to comprehensively investigate the mRNA expression levels of proinflammatory cytokines, inflammatory mediators, nerve growth factor and its receptor, and matrix metalloproteinases in the synovium of dogs with spontaneous OA as well as to elucidate their relationships with the severity of synovitis. Dogs that were diagnosed with stifle OA on the basis of radiographic findings were included, and the degree of synovitis was observed using stifle arthroscopy. The dogs were assigned to two different groups depending on their synovitis scores: the low-grade group (score of 1 or 2; n = 8) and high-grade group (score of 3 to 5; n = 18). The dogs showing no evidence of orthopedic disease were included in the control group (n = 6). Synovial tissue samples were collected from the sites at which synovitis scores were assessed using arthroscopy. Total RNA was extracted from the collected synovial tissue, and cDNA was synthesized. Subsequently, RT-qPCR were performed using canine-specific primer sets for *IL1B*, *IL6*, *CXCL8*, *TNF*, *TGFB1*, *PTGS2*, *PTGES*, *MMP3*, *MMP13*, *NGF*, *NTRK1*, and *PTGER4*. Expression levels of *IL1B*, *IL6*, *CXCL8*, and *MMP13* were significantly higher in the high-grade group than in the control group. In addition, expression levels of *IL1B*, *CXCL8*, *TNF*, and *PTGS2* were significantly higher in the high-grade group than in the low-grade group. Expression levels of *IL1B*, *IL6*, *CXCL8*, *TNF*, *PTGS2*, and *PTGER4* showed significant positive correlation with synovitis score. In conclusion, all mRNA expression levels in the synovial membrane varied according to the degree of synovitis in dogs with spontaneous OA. Thus, this study may partially elucidate the pathogenesis of synovitis in dogs with spontaneous OA.

## Introduction

Canine osteoarthritis (OA) is more prevalent because of the prolongation dogs’ lifetime with the advancement of veterinary medicine. One report estimated that approximately 20% of adult dogs in the United States are affected with OA [1]. Although OA is one of the major causes of chronic pain in dogs, the pathogenesis of OA has not been fully understood.

OA is a disorder of synovial joints characterized by aberrant repair and eventual degeneration of articular cartilage as well as by the formation of new bone at the articular margins, sclerosis of subchondral bone, and development of synovial inflammation of variable grade [2]. In human OA, products of cartilage breakdown released into the synovial fluid are phagocytosed by synovial cells, and this process amplifies synovial inflammation. In turn, activated synovial cells in the inflamed synovium produce inflammatory mediators such as interleukin (IL)-1β, IL-6, IL-8, tumor necrosis factor-α (TNF-α), cyclooxygenase-2 (COX-2), prostaglandin E_2_ (PGE_2_), and leukotriene B4 (LTB_4_). In addition, these inflammatory mediators lead to excessive production of the proteolytic enzymes such as matrix metalloproteinases (MMPs) and a disintegrin and metalloproteinase with thrombospondin motifs (ADAMTS) responsible for cartilage breakdown. Furthermore, the inflamed synovium contributes to the formation of osteophytes via transforming growth factor-β (TGF-β) and bone morphogenetic proteins (BMPs) [3]. It is assumed that this pathogenesis of OA is similar in dogs.

Synovitis occurs from a relatively early stage, and clinical signs such as joint pain are associated with the degree of synovitis in human OA cases [4]. Therefore, there have been a large number of studies on pathogenesis of OA-related synovitis in humans. On the contrary, most of the studies on canine OA have focused on the degeneration and degradation of articular cartilage; however, only a few have focused on the synovial inflammation. In dogs with experimentally induced OA, the mRNA expression levels of IL-1β, MMP-3, and collagenase increased in the synovial membrane of affected stifle joint [5–7]. To the best of our knowledge, there has been only one report that investigated the mRNA expression levels of inflammatory cytokines in the synovial membrane collected from the joint affected by spontaneous OA in dogs [8]. The report demonstrated that the mRNA expression levels of IL-1β, IL-6, and IL-10 were significantly higher in dogs with spontaneous OA than in healthy dogs [8]. Thus, studies on the mRNA expression levels and patterns of proinflammatory cytokines in the synovial membrane of affected joints are overwhelmingly scarcer in relation to dogs with spontaneous OA than in relation to humans with OA. Furthermore, variations in mRNA expression levels of proinflammatory cytokines and mediators according to the severity of synovitis has not been determined.

In recent years, prostaglandin E receptor 4 (EP4) inhibitor and anti-nerve growth factor (NGF) antibody have been used as novel treatments for OA in dogs [9, 10]. However, no report revealed that the mRNA expressions of EP4, NGF, and tropomyosin receptor kinase A (TrkA) as high-affinity NGF receptor in the synovial membrane of the affected joint in dogs with spontaneous OA. Therefore, it is necessary to reveal the expression levels and patterns of proinflammatory cytokines and mediators in the synovial membrane to further understand the pathogenesis and to develop new treatments for OA in dogs.

The objective of this study was to comprehensively investigate the mRNA expression levels of proinflammatory cytokines and a growth factor (IL-1β, IL-6, IL-8, TNF-α, and TGF-β), inflammatory mediators (COX-2, prostaglandin E synthase, PGES and EP4), nerve growth factor and its receptor (NGF and TrkA), and matrix metalloproteinases (MMP-3 and MMP-13) in the synovial membranes of dogs with spontaneous OA as well as to elucidate their relationships with the severity of synovitis.

## Materials and methods

### Dogs and samples

**Fig 1:**
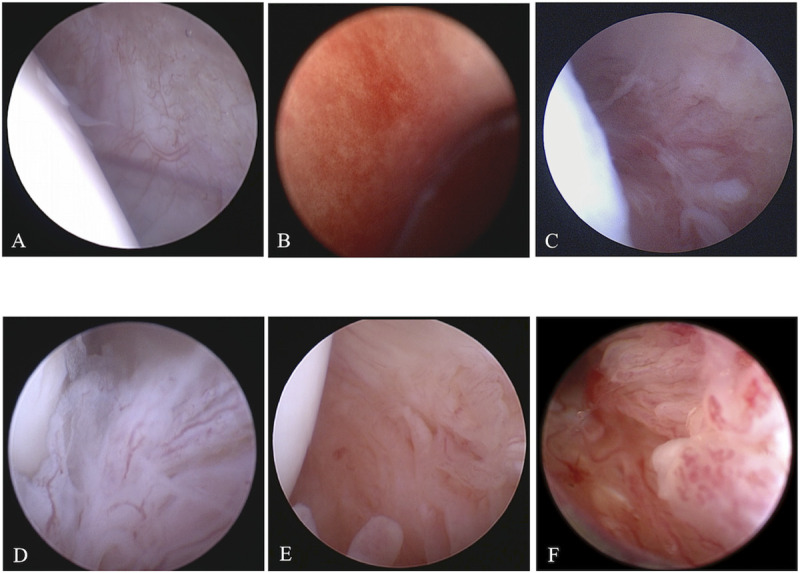
Representative arthroscopic images for each synovitis grade. (A) Grade 0: smooth with sparse well defined blood vessels, (B) Grade 1: focal involvement and notable increase in vascularity, (C) Grade 2: visible proliferation/fimbriation/thickening and notable vascularity, (D) Grade 3: consistent notable proliferation/fimbriation/thickening and moderate vascularity, (E) Grade 4: consistent and marked proliferation/fimbriation/thickening and diffuse hypervascularity, (F) Grade 5: consistent and severe proliferation/fimbriation/thickening and severe hypervascularity.

In the present study, dogs that were presented to the Animal Medical Center of Nihon University between January 2019 and July 2020 and were diagnosed with stifle OA on the basis of their radiographic findings were included. This study was approved by the clinical research and trial ethics committee, Animal Medical Center, Nihon University (ANMEC-3-005). In all cases, the degree of synovitis was determined using stifle arthroscopy, and synovitis was graded on a scale of 0 to 5 as described in a previous study [11] (Fig 1). In the present study, the dogs with a synovitis grading score of 1 or 2 were assigned to the low-grade group, and the dogs with a synovitis grading score of 3 to 5 were assigned to the high-grade group. Synovial tissue samples were then collected from the sites at which synovial scores were assessed using arthroscopy. The collected synovial tissue samples were immediately frozen using liquid nitrogen and stored in a deep freezer at −80°C until the extraction of RNA.

In the present study, the dogs showing no evidence of orthopedic disease were assigned to the control group. This study was approved by the Nihon University Animal Use and Care Committee (AP19BRS066-1). A small incision of approximately 1 cm was made in the stifle joint under general anesthesia, and synovial tissue samples were collected after confirming that there was no evidence of synovitis. These dogs were received appropriate post-operative management including administration of analgesics and antibiotics according to institutional regulations. The collected tissue samples were stored as described above.

### Total RNA extraction

Frozen stored synovial tissue samples were crushed in liquid nitrogen, immediately dissolved in 1 ml of TRIzol reagent (Thermo Fisher Scientific Inc., Waltham, MA, U.S.A.), and homogenized. Subsequently, 200μl of chloroform was added to the homogenized tissue solution and centrifuged at 12,000 g and 4°C for 15 minutes. Total RNA was extracted using miRNeasy Mini kit (QIAGEN, Hilden, Germany), and DNase treatment was performed using RNase-Free DNase set (QIAGEN). Finally, total RNA was eluted in 30 μl of RNase-free water. The concentration and 260/280 ratio of total RNA were measured using ND-1000 (Thermo Fisher Scientific Inc.).

### cDNA synthesis

Reverse transcription was performed using PrimeScript RT master mix (TaKaRa Bio Inc., Kusatsu, Shiga, Japan) and My Genie 32 Thermal Block (BIONEER Co., Daejon, Korea). To describe the method briefly, 500ng of total RNA was mixed with 4 μl of PrimeScript RT master mix, and RNase-free water was added to this solution to bring the total volume to 20 μl. Subsequently, the reverse transcription reaction was performed at 37°C for 15 minutes, and the reverse transcriptase was inactivated by incubation at 85°C for 5 seconds. The cDNA samples were stored in a deep freezer at -80°C until quantitative real-time reverse transcriptase PCR (RT-qPCR) was performed.

### RT-qPCR assay

RT-qPCRs were performed in duplicate with 1 μ*l (template*: *12*.*5ng)* of the first-strand cDNA using canine-specific primer sets (TaKaRa Bio Inc.) for *IL1B*, *IL6*, *CXCL8*, *TNF*, *TGFB1*, *PTGS2*, *PTGES*, *MMP3*, *MMP13*, *NGF*, *NTRK1*, and *PTGER4* (Table 1) and TB Green Premix Ex Taq II (TaKaRa Bio Inc.) using Thermal Cycler Dice^®^ Real Time System II (TaKaRa Bio Inc.). These canine-specific primers were designed using NCBI Primer-BLAST. Each PCR involved 1 cycle of denaturing at 95°C for 30 seconds, 40 cycles of denaturing at 95°C for 5 seconds, and annealing and extension at 60°C for 30 seconds. The results were analyzed using the crossing point method and the comparative cycle threshold (ΔΔCt) method with TP900 DiceRealTime v4.02B (TaKaRa Bio Inc.). RT-qPCR of no-template controls was performed with 1 μl RNase- and DNA-free water, and amplification of *GAPDH* from the same amount of cDNA was used as an endogenous control. The specificity of the amplified PCR products was verified by analysis of the melting curve. The relative expression levels of each gene were compared among the three groups: control, low-grade, and high-grade groups. Furthermore, the correlation between the relative expression level of each gene and synovitis score was also investigated.

**Table 1:** Canine-specific primer for RT-qPCR.

Name	Gene symbol	NCBI accession number	Forward primers (5' - 3')	Reverse primers (5' - 3')	Product (bp)	Location
GAPDH	*GAPDH*	NM_001003142.2	GATGGGCGTGAACCATGAGA	AGTGGTCATGGATGACTTTGGCTA	107	452–558
IL-1β	*IL1B*	NM_001037971.1	GCCAAGACCTGAACCACAGT	CTGACACGAAATGCCTCAGA	96	107–202
IL-6	*IL6*	NM_001003301.1	ACCGGTCTTGTGGAGTTTCA	CAGGATCTTGGTACTCATGTGC	102	391–492
IL-8	*CXCL8*	NM_001003200.1	TTCAGAACTTCGATGCCAGT	GGGCCACTGTCAATCACTCT	90	93–182
TNF-α	*TNF*	NM_001003244.4	GAGCACTGAAAGCATGATCC	GAGAAGAGGCTGAGGCAGAA	108	3–110
TGF-β	*TGFB1*	NM_001003309.1	CCCTGGACACCAACTACTGCTTC	GGATCCACTTCCAGCCCAGA	99	893–991
COX-2	*PTGS2*	NM_001003354.1	GATCATAAGCGAGGACCAGCTTTC	GGCGCAGTTTATGTTGTCTATCCA	100	623–722
PGES	*PTGES*	NM_001122854.1	GTATTGCCGGAGTGACCAGGA	AGTGCATCTGGGCGATGAAAG	136	232–368
MMP-3	*MMP3*	NM_001002967.1	CCTAGCGCTCTGATGTACCC	GGACTGGATGCCATTCACAT	90	718–807
MMP-13	*MMP13*	XM_022418390.1	AGTTCGGCCACTCCTTAGGT	CATCGGGAAGCATAAAGTGG	102	1692–1793
NGF	*NGF*	NM_001194950.1	CACTGGACTAAGCTTCAGCATTC	TGTCACCCTGGCAGCTATTG	87	106–192
TrkA	*NTRK1*	XM_022421240.1	CCAGGGTGATCTCAAAGCATCTAAC	CCATCTAGCGAGGCAGGAACA	94	2504–2597
EP4	*PTGER4*	NM_001003054.1	TCCAGATGGTCATCCTGCTCA	GGATAGGGTTCACAGCAGCAA	169	1101–1269

### Statistical analysis

The data obtained from the described experiments were calculated as mean ± standard deviation. Statistical analyses were performed using GraphPad Prism version 6.0 for Macintosh (GraphPad Software Inc., San Diego, CA, U.S.A.). Kruskal-Wallis test was used for comparisons among the three groups, and Dunn’s test was used for post-hoc comparisons. Spearman’s rank correlation coefficients were calculated to assess the correlation between the relative expression level of each gene and synovitis score. Significance threshold was set at *p* < 0.05.

## Results

### Dogs

In the control group, the synovial tissue samples were collected from 6 stifle joints of 4 normal dogs. In this group, the mean age and body weight were 6.6 ± 5.2 years (range, 2.1 to 11.1 years) and 10.5 ± 0.6 kg (range, 9.9 to 11.1 kg), respectively. Two each of male and female dogs were included, and all dogs were Beagles.

In the low-grade group, synovial tissue samples were collected from 8 stifle joints of 8 dogs with spontaneous OA. In this group, the mean age and body weight were 5.4 ± 4.5 years (range, 1.1 to 11.8 years) and 10.7 ± 12.3 kg (range, 1.7 to 36.5 kg), respectively. In this group, one male dog (castrated) and seven female dogs (1 intact and 6 spayed) were included. Breeds included were Mix (n = 3), Bernese Mountain Dog (n = 1), Chihuahua (n = 1), Pekingese (n = 1), Shiba (n = 1), and Toy Poodle (n = 1). Six stifle joints had OA owing to patellar luxation, and the remaining two had OA owing to the rupture of cranial cruciate ligament.

In the high-grade group, synovial tissue samples were collected from 18 stifle joints of 18 dogs with spontaneous OA. In this group, the mean age and body weight were 6.5 ± 3.0 years (range, 0.5 to 12.1 years) and 21.0 ± 11.4 kg (range, 4.2 to 46.0 kg), respectively. Six male dogs (1 intact and 5 castrated) and 12 female dogs (1 intact and 11 spayed) were included. Breeds included were Golden Retriever (n = 3), Mix (n = 3), Toy Poodle (n = 3), Bernese Mountain Dog (n = 2), Akita (n = 1), Border Collie (n = 1), English Setter (n = 1), Jack Russell Terrier (n = 1), Jindo dog (n = 1), Labrador Retriever (n = 1), and Pembroke Welsh Corgi (n = 1). Sixteen stifle joints had OA owing to the rupture of cranial cruciate ligament, and the remaining two had OA owing to patellar luxation.

### Gene expression levels associated with synovitis

**Fig 2:**
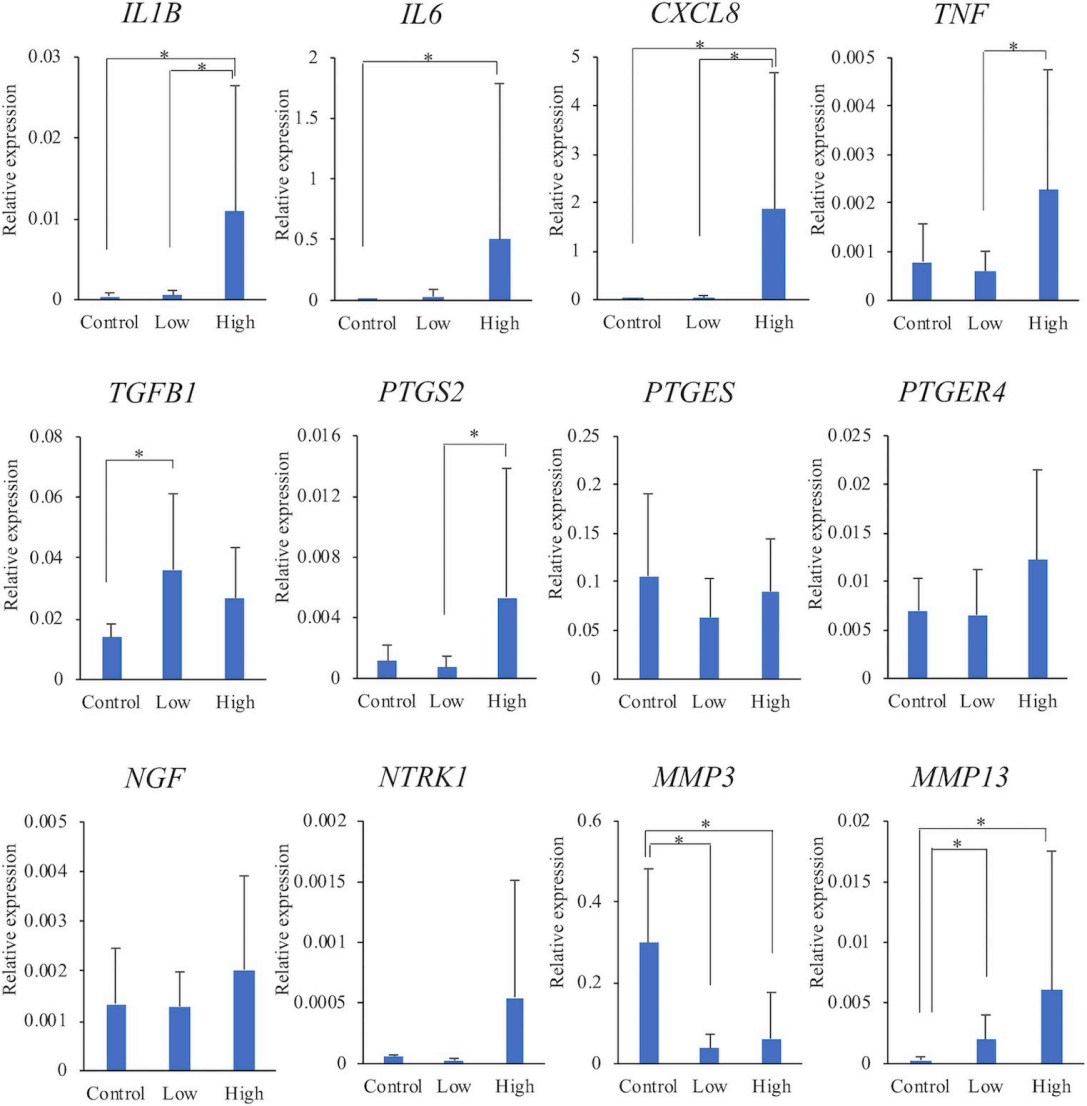
Comparison of mRNA expression levels among the three groups. The relative expression levels of each gene were compared among the three groups. The data are presented as mean ± standard deviation. Low: low-grade group, High: high-grade group. *: Mean values differ significantly between the groups (*p* < 0.05).

The gene expression levels in each group are shown in Fig 2. When the control group was compared with the low-grade group, there were no significant differences in the expression levels of *IL1B*, *IL6*, *CXCL8*, *TNF*, *PTGS2*, *PTGES*, *PTGER4*, *NGF*, and *NTRK1* between the groups. Expression levels of *TGFB1* and *MMP13* were significantly higher in the low-grade group than in the control group. On the contrary, the expression level of *MMP3* was significantly lower in the low-grade group than in the control group.

Expression levels of *IL1B*, *IL6*, *CXCL8*, and *MMP13* were significantly higher in the high-grade group than in the control group. Expression levels of *TNF*, *PTGS2*, *PTGER4*, *NGF*, and *NTRK1* also tended to be higher in the high-grade group than in the control group. On the contrary, the expression level *MMP3* was significantly lower in the high-grade group than in the control group.

With regard to comparison of the low-grade and high-grade groups with different grades of synovitis, expression levels of *IL1B*, *CXCL8*, *TNF*, and *PTGS2* were significantly higher in the high-grade group than in the low-grade group. In addition, expression levels of *IL6*, *PTGER4*, *NGF*, *NTRK1*, and *MMP13* tended to be higher in the high-grade group than in the low-grade group.

### Correlation between the gene expression levels and synovitis scores

**Fig 3:**
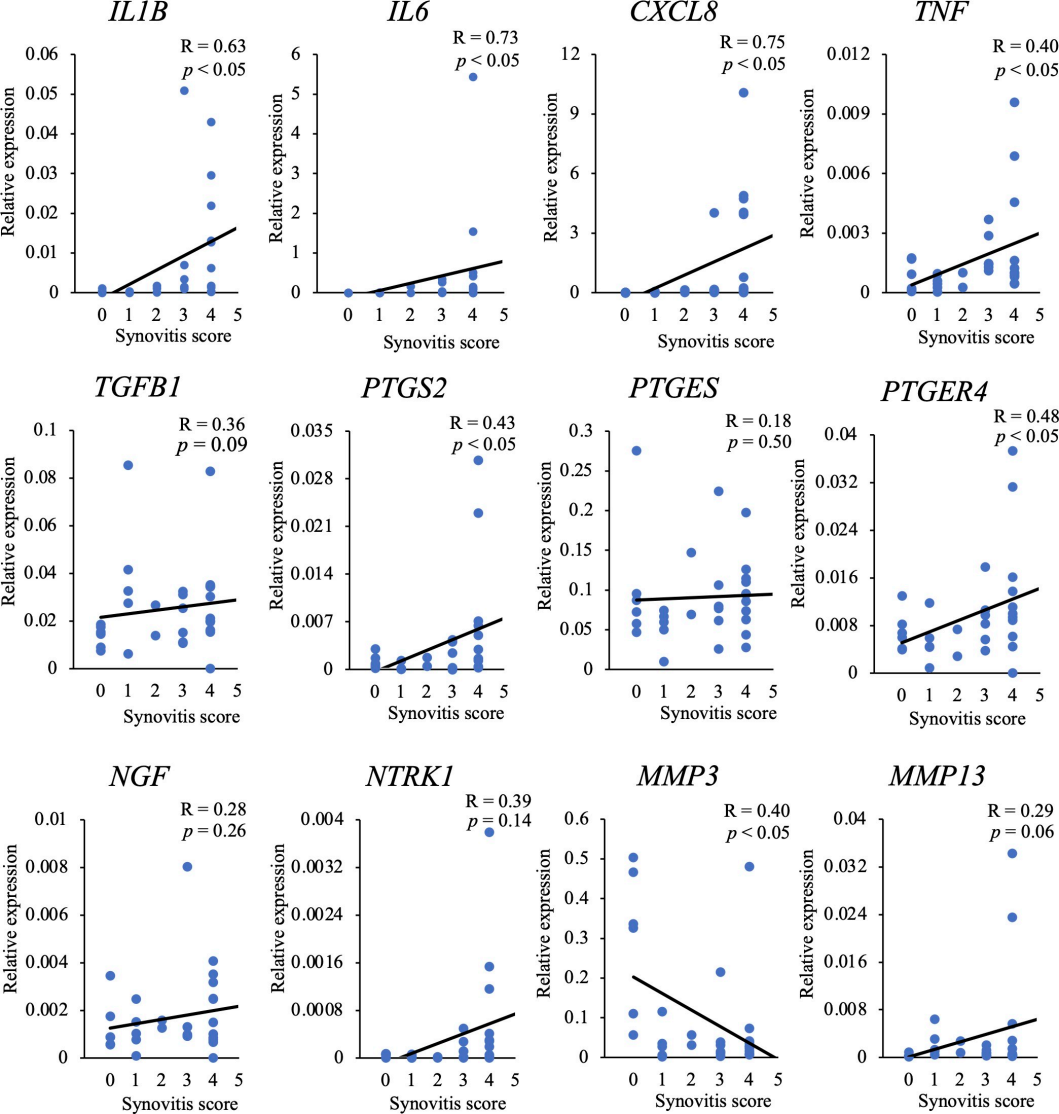
Correlation between mRNA expression levels and synovitis scores. The correlation between the relative expression level of each gene and synovitis score was investigated. R: correlation coefficient.

Correlations between the gene expression levels and synovitis scores are shown in Fig 3. Expression levels of *IL1B*, *IL6*, *CXCL8*, *TNF*, *PTGS2*, and *PTGER4* showed significant positive correlations with synovitis score. In addition, the expression level of *NTRK1* tended to increase with an increase in the score. On the contrary, there was a significantly negative correlation between the expression level of *MMP3* and synovitis score.

## Discussion

The present study revealed an insight into the pathogenesis of synovitis by conducting a comprehensive investigation on the gene expression levels involved in inflammation of the synovium in dogs with spontaneous OA and their associations with the severity of synovitis. In addition, this is the first report on the mRNA expression levels of NGF, TrkA, and EP4 in the synovium of dogs with spontaneous OA.

Proinflammatory cytokines and growth factor such as IL-1β, IL-6, IL-8, TNF*α*, and TGF-β are an important group of mediators participating in the pathogenesis of OA in humans. These cytokines and growth factor play an important role in increasing inflammation of the synovial membrane and the formation of osteophytes [12–14]. Previous studies reported that levels of IL-1β, IL-6, IL-8, and TNF-α were elevated in the synovium of humans with OA [12, 13] and that IL-1β induced a significant increase in the mRNA expression levels of these cytokines in synovial fibroblasts of patients with OA [15]. Another study demonstrated that TGF-β in the synovium of humans with OA was increased with an increase in radiography-based OA severity [14]. In the present study, expression levels of *IL1B*, *IL6*, *CXCL8*, and *TGFB1* were significantly higher in either of OA groups than in the control group. In addition, all mRNA expression levels related to the proinflammatory cytokines, except *TGFB1*, increased significantly with an increase in the synovitis score. Thus, the present study revealed that expression patterns of *IL1B*, *IL6*, *CXCL8*, *TNF*, and *TGFB1* in the synovium of dogs with spontaneous OA were similar to those of humans with OA.

One of the first effects of proinflammatory cytokines in patients with OA is the activation of phospholipase A2, which cleaves cellular membranes, thereby liberating arachidonic acid [16]. Arachidonic acid is then converted into prostaglandin H_2_ (PGH_2_), and this conversion is mediated by COXs. Among the COXs, COX-2 is more efficient at producing prostaglandins than is COX-1 [17] and is considered to be associated mainly with inflammatory events of OA. PGH_2_ is subsequently converted into PGE_2_ by PGES. PGE_2_ exerts its effects via four receptors and one of them, i.e., EP4 receptor, is primarily responsible for the pain and inflammation associated with OA [10, 18]. Thus, NSAIDs that suppress COX-2 and reduce the production of prostaglandins have been widely used to treat joint pain in dogs. In addition, EP4 inhibitor as a new type of anti-inflammatory analgesic for canine OA was launched in the United States in 2017 and Europe in 2019. In the present study, *PTGS2*, *PTGES*, and *PTGER4* expression levels in the synovium of canine spontaneous OA were investigated because the mRNA expressions of prostaglandins cannot be evaluated directly. We found that expression levels of *PTGS2* and *PTGER4* were increased significantly with an increase in the synovitis score. A previous study reported that the expression level of *PTGS2* in the synovium of humans with OA was higher than that in the synovium of normal humans [19]. *In vitro* studies using human synovial fibroblasts demonstrated that the stimulation by IL-1β increased the expression level of *PTGER4* [20]. The present study revealed that *PTGS2* and *PTGER4* expression patterns in the synovia of dogs with spontaneous OA were similar to those in the synovia of humans. In addition, the results of this study suggest that EP4 inhibitors as well as NSAIDs may be effective in the treatment of severe OA-induced synovitis in dogs.

NGF is produced by stimulation of proinflammatory factors such as IL-1β and TNF-α in synovia with OA [13, 21]. NGF functions as a soluble signaling protein that mediates its activity via binding to the high-affinity NGF-specific TrkA and contributes to OA pathology and joint pain [21]. A previous study reported that NGF expression level was higher in synovia of humans with OA than in normal humans [22]. *In vitro* studies using human synovial fibroblasts demonstrated that the stimulation by IL-1β increased NGF levels and the addition of NGF increased TrkA levels [23]. In the present study, expression levels of *NGF* and *NTRK1* tended to be high, especially in the high-grade group. In addition, the expression level of *NTRK1* tended to increase with an increase in the score. Thus, the results of this study suggest that levels of NGF and its receptor increased depending on the severity of synovitis in the synovium of dogs with spontaneous OA, as in humans with OA. These results support the effectiveness of the use of anti-NGF antibodies for the treatment of severe OA-induced synovitis in dogs.

Proinflammatory cytokines and inflammatory mediators induce excessive production of MMPs, and large quantities of MMPs are produced by synovial cells and chondrocytes [13]. Among the MMPs, MMP-3 and MMP-13 are considered to be the major catabolic effectors in OA [24]. Previous studies reported that MMP-3 levels and mRNA expression levels of MMP-13 were higher in synovia of humans with OA than in normal humans [25, 26]. In the present study, *MMP3* expression levels in both the low- and high-grade groups were significantly lower than those in the control group. These results differed from those of the human studies; however, there has been shown that MMP-3 levels were significantly lower in the synovial fluid of dogs with spontaneous OA than that of normal dogs [27]. Therefore, a part of the pathology in OA may be different between human and dogs. On the contrary, the present study revealed that *MMP13* expression levels in the synovia of dogs with OA were similar to those in the synovia of human with OA. Further studies are needed to determine the relationship between the mRNA expression level and role of MMPs in canine OA.

The limitations of this study included the small numbers of individuals each group. All controls were smaller in size than non-controls and composition of breed and gender was different among the three groups. In addition, only Beagles were used as controls. Further studies including other breeds may be needed to eliminate breed-specific gene expression patterns. In the present study, *PTGES* expression level was assessed as an indicator of PGE_2_; however, further studies using ELISA are needed to directly examine the extent of PGE_2_ release from the synovia of dogs with OA. In the present study, all types of OA in the human studies referenced were primary OA, which is different from secondary OA in dogs. Therefore, some of the differences in inflammation patterns between human and dogs may be due to the inciting pathogenesis. Further, the cases of patellar luxation and rupture of cranial cruciate ligament was more prevalent in the low-grade group and in the high-grade group. This may have reflected the more severe synovitis occurred in the case with rupture of cranial cruciate ligament than with patellar luxation. Therefore, further investigations with a higher number of cases of severe synovitis owing to patellar luxation are needed.

## Conclusion

The mRNA expression levels of proinflammatory cytokines, inflammatory mediators, NGF and its receptor, and MMPs in the synovial membrane varied according to the degree of the synovitis in dogs with spontaneous OA. Thus, this study may partially elucidate the pathogenesis of synovitis in dogs with spontaneous OA and may contribute to the development of new treatments for OA in dogs.
